# Reviews

**Published:** 1862-01

**Authors:** 


### REVIEWS

*Lectures on Materia Medica and Therapeutics, delivered in the College of Physicians and Surgeons of the University of New York. By* JOHN B. BECK, M. D., *late Professor of Materia Medica and Medical Jurisprudence. Prepared for the Press by his friend,* C. R. GILMAN, M. D., *Professor of Obstetries, etc., in the College of Physicians and Surgeons, N. Y. Third Edition. New York:* SAMUEL S. & WILLIAM WOOD, 33 *Broadway*, 1861.

That this book should continue *the* text book of materia medica for so many years, is proof that our science is becoming settled, and in a good degree certain. This last edition of Beck's Materia Medica, commends itself not only to medical students, but also to physicians; not that new remedies are presented and urged upon the consideration of the reader, but since the *old* and truly valuable medicines are faithfully described, and new *principles* of therapeutics established, while the best means of bringing those principles into practice are fully and clearly presented. The character and value of this book is too well established to admit of any doubt as to the high estimation in which it is universaly held by those who are acquainted with its merits. It contains a full, clear, and comprehensive description of all remedies of established value, and the therapeutical principles which govern their administration.

For sale in this city by THEODORE BUTLER, No. 159 Main street.

---

*Lectures on the Diseases of Women, by* CHARLES WEST, M. D., *Fellow of the Royal College of Physicians; Examiner in Midwifery at the Royal College of Surgeons of England; Physician Accoucher to St. Bartholomew's Hospital; and Physician to the Hospital for sick Children; author of "Lectures on the Diseases of Infancy and Childhood." Second American, from the Second London Edition Philadelphia:* BLANCHARD & LEA, 1861.

The interest and importance which is now universally attached to the diseases peculiar to women, will render this new volume by Dr. West. indispensabie. The intelligent women of our country have learned enough of themselves and of the diseases to which they are peculiarly exposed, together with the expedients which have been made available for their relief, to longer remain satisfied with the attendance of a physician who does not show some familiarity with the proper modes of investigation and some aptness in directing efficient means for their relief. The day has nearly passed when a physician can safely forget, in the treatment of diseases occurring among patients of the female sex, that, besides the ordinary causes of disease common to both sexes, there is another set of causes peculiar to women.

The disorders of the sexual functions and the way in which they re-act upon the general health, or are acted upon by it, manifestly deserve the most careful attention, since it is often no easy task to unravel the tangled web of symptoms and find out where the mischief is, which has given rise to so various manifestations of diseases. To say that a female patient has neuralgia, spinal irritation, or hysteria, prescribing valerian assafœtida or counter irritation, and neglecting to institute thorough investigation into the more primary causes of disease, constitutes the grossest stupidity.—But this is not what we designed to say, and has only been said accidentally, while thinking of the light which has been shed upon these topics by Dr. West and others within the last few fears, and remembering how few, comparatively, had ever caught the first gleam of this sunlight. West on the Diseases of Females, should be placed upon the table of every practicing physician—should be read and carefully considered. It contains thirty-three lectures, and treats upon the diseases of women *rationally.*

